# Interaction between ABA and NO in plants under abiotic stresses and its regulatory mechanisms

**DOI:** 10.3389/fpls.2024.1330948

**Published:** 2024-05-17

**Authors:** Junrong Xu, Xuefang Lu, Yunzhi Liu, Weisen Lan, Zhien Wei, Wenjin Yu, Changxia Li

**Affiliations:** College of Agriculture, Guangxi University, Nanning, China

**Keywords:** environmental stresses, regulatory pathways, metabolic pathways, abscisic acid, nitric oxide, crosstalk

## Abstract

Abscisic acid (ABA) and nitric oxide (NO), as unique signaling molecules, are involved in plant growth, developmental processes, and abiotic stresses. However, the interaction between ABA and NO under abiotic stresses has little been worked out at present. Therefore, this paper reviews the mechanisms of crosstalk between ABA and NO in the regulation of plants in response to environmental stresses. Firstly, ABA-NO interaction can alleviate the changes of plant morphological indexes damaged by abiotic stresses, for instance, root length, leaf area, and fresh weight. Secondly, regulatory mechanisms of interaction between ABA and NO are also summarized, such as reactive oxygen species (ROS), antioxidant enzymes, proline, flavonoids, polyamines (PAs), ascorbate-glutathione cycle, water balance, photosynthetic, stomatal movement, and post−translational modifications. Meanwhile, the relationships between ABA and NO are established. ABA regulates NO through ROS at the physiological level during the regulatory processes. At the molecular level, NO counteracts ABA through mediating post-translational modifications. Moreover, we also discuss key genes related to the antioxidant enzymes, PAs biosynthesis, ABA receptor, NO biosynthesis, and flavonoid biosynthesis that are regulated by the interaction between ABA and NO under environmental stresses. This review will provide new guiding directions for the mechanism of the crosstalk between ABA and NO to alleviate abiotic stresses.

## 1 Introduction

Abiotic stresses such as drought, salt, heavy metal, extreme light, ultraviolet-B (UV-B), and extreme temperature are usually caused by changes in environmental factors (Gujjar et al., 2014; Kreszies et al., 2018; Stromme et al., 2019). When plants are exposed to extreme environments, it often results in morphological and physiological changes, for instance, growth inhibition (Pontin et al., 2021), chlorophyll degradation (Sahay et al., 2019), stomatal closure (Peng et al., 2022), and redox imbalance (Lu et al., 2009). In response to these adverse effects, plants often stimulate reactive oxygen species (ROS) generation, including hydrogen peroxide (H_2_O_2_), superoxide radicals (O_2_^-^), and hydroxyl radicals (OH^–^). Interestingly, ROS is considered a double-edged sword: promotion of plant growth at low concentrations and inhibition at high levels (Li et al., 2022). When the stress exceeds the tolerance capacity of plants, excess ROS is produced in plant cells (Gill and Tuteja, 2010; Mullineaux et al., 2018; Zhang et al., 2018a). High levels of ROS lead to excessive oxidation of the plasma membrane, frequently producing high levels of malondialdehyde (MDA) and thiobarbituric acid reactive substances (TBARS), resulting in plant cell poisoning (Xu et al., 2013; Berni et al., 2019; Iqbal et al., 2022). To scavenge excess ROS, plants can regulate antioxidant enzymes, antioxidants, phytohormones, and signaling molecules in response to stress (Muneer et al., 2014; Peng et al., 2022; Saha et al., 2022). Moreover, abscisic acid (ABA) and nitric oxide (NO) significantly enhance antioxidant enzyme activities to eliminate excess ROS, thus increasing the survival percentage of maize seedlings under chilling stress (Li and Zhang, 2012). The polyethylene glycol (PEG)-induced reduction in length, fresh weight, and dry weight of roots are reversed by exogenous ABA and NO (Sahay and Gupta, 2018; Sahay et al., 2019). The authors also found that ABA and NO can increase the levels of antioxidant enzymes and reduce ROS and MDA contents (Sahay and Gupta, 2018; Sahay et al., 2019). The above studies imply that ABA and NO play a crucial role in inhibiting ROS and reducing plasma membrane oxidation under abiotic stresses.

ABA is an essential plant hormone, which is closely linked to seed germination (Kermode, 2005), stomatal closure (Raschke et al., 1975), and defoliation (Gao et al., 2017), especially abiotic stresses (Sah et al., 2016). It has been reported that ABA regulates stomatal movement and abscission by binding to specific receptors under abiotic stresses (Iqbal et al., 2021). Meanwhile, the regulatory mechanisms of ABA under stresses are complex, because ABA interacts with other signaling molecules, such as ROS, calcium ion (Ca^2+^), hydrogen sulfide (H_2_S), and NO (Desikan et al., 2004). As a signaling molecule, NO is involved in chlorophyll biosynthesis (Zhang et al., 2018b), flowering time (Grun et al., 2006), crop yield (Hou and Tsuruta, 2003), stomatal movement (Castillo et al., 2015), and abiotic stresses (Iqbal et al., 2022). Simultaneously, NO also acts in conjunction with other signaling molecules or hormones under abiotic stresses. For instance, our previous research has shown that NO is involved in melatonin-induced antioxidant response in the leaves of tomato seedlings under cadmium stress (Xu et al., 2023). Moreover, NO modulates seed germination, stomatal movement, and environmental tolerance by post-translational modifications (PTMs), including S-nitrosylation and tyrosine nitration (Albertos et al., 2015; Wang et al., 2015; Yang et al., 2015). For S-nitrosylation, it is produced by covalent binding between the NO group and thiol (–SH) of cysteine (Prakash et al., 2019; Iqbal et al., 2021). In addition, susceptible amino acids are bonded with a nitro group to develop tyrosine nitration (Prakash et al., 2019; Iqbal et al., 2021).

A growing number of reports indicate that the interaction between ABA and NO participates in abiotic tolerance (Zhang et al., 2019; Santos et al., 2020; Iqbal et al., 2022). For example, the growth of wheat is altered by ABA and NO under heat stress, including increase of the length, fresh weight, dry weight, and leaf area (Iqbal et al., 2022). ABA and NO solution treatments can maintain firmness and peel color as well as improve antioxidant capacity in peach fruit under low-temperature stress, however, ABA-induced cold tolerance is blocked by NO scavenger 2-(4-carboxyphenyl)-4,4,5,5-tetramethylimidazoline-1-oxyl-3 oxide (cPTIO) (Zhang et al., 2019). Zhang et al. (2018b) imply that low-light stress can cause a decrease in plant height, leaf width, tiller number, and dry weight of tall fescue seedlings, whereas ABA alleviates the reduction, following an increase in endogenous NO concentration. Interestingly, ABA biosynthesis inhibitor fluridone (Flu) can suppress endogenous NO generation. These results indicate that ABA promotes plant growth by increasing NO response under low-light stress. Under chilling stress, ABA and NO donor sodium nitroprusside (SNP) enhance the activities of antioxidant enzymes to reduce ROS levels in maize seedlings (Li and Zhang, 2012). Nevertheless, ABA-activated antioxidant responses are reversed by NO scavenger cPTIO, suggesting that ABA enhances chilling tolerance relying on NO.

The above results show that crosstalk between ABA and NO is involved in abiotic tolerance, but few reviews have summarized and discussed it. Thus, this paper presents a comprehensive review of the interaction between ABA and NO under abiotic tolerance and their regulatory pathways. In addition, the expressions of key genes mediated by interaction between ABA and NO are briefly discussed.

## 2 ABA-NO interaction confers tolerance to plants under environmental stresses

### 2.1 Drought

Drought stress affects the growth, development, and survival of plants (Wang et al., 2018). However, it has been shown that ABA, NO, and/or ROS contribute/contributes to stress mitigation. For example, the roots tips of wheat seedlings are detached, then the shed root tips are exposed to drought stress by evaporating (Zhao et al., 2001). The activities of superoxide synthase and NO synthase-like (NOS-like, EC 1.14.13.39) are enhanced at 20 min after drought treatment, and the content of endogenous ABA is accumulated after 60 min. It is interesting that ABA-induced drought stress response is inhibited by ROS scavenger, NOS-like inhibitor, or NO scavenger, indicating that ROS and NO are involved in ABA-induced drought tolerance in root tips of wheat seedlings (Zhao et al., 2001; **Table 1**). Tari et al. (2010) reveal that the root length of drought-tolerant wheat cultivar is lower than that of drought-sensitive wheat cultivar under drought stress. Meanwhile, high levels of ROS, ABA, and NO are detected in drought-tolerant wheat cultivar, showing that the generations of ROS and NO are induced by ABA under osmotic stress. Under osmotic tolerance, the leaf water loss is reduced by SNP and H_2_O_2_ in wheat seedlings (Xing et al., 2004; **Table 1**). Meanwhile, SNP and H_2_O_2_ can improve ABA content during the process. However, the effects are reversed by NO scavenger and NOS inhibitors. The results demonstrate that there is a crosstalk between ABA and NO under drought stress. Exogenous ABA can improve drought tolerance by regulating antioxidant systems and relative water content (RWC) as well as generating NO and H_2_O_2_ in triploid bermudagrass (Lu et al., 2009). The authors further found that NO and H_2_O_2_ also activate antioxidant systems in response to drought stress (Lu et al., 2009). Moreover, ABA-induced drought tolerance is reduced by NO scavenger/inhibitor and H_2_O_2_ scavenger/inhibitor. Furthermore, H_2_O_2_ scavenger/inhibitor can suppress NO-induced drought tolerance. The above results show that ROS is linked between ABA and NO under drought stress. For tomato seeds under osmotic stress, 3 μM ABA accelerates seed dormancy, and NO donor S-nitrosoglutathione (GSNO) can reverse the effects (Piterkova et al., 2012). Endogenous ABA, NO, H_2_O_2_ in the leaves of *Bromeliaceae* are accumulated, and RWC is reduced during drought stress, suggesting that ABA, NO, and H_2_O_2_ play an important role in the response to drought stress by osmotic adjustment (Mioto and Mercier, 2013; **Table 1**). Planchet et al. (2014) report that PEG-induced accumulation of NO in *Medicago* seedlings is reduced by the ABA biosynthesis inhibitor norflurazon. Interestingly, under normal conditions, NO content is increased by exogenous ABA, but this effect is reversed by NO scavenger cPTIO (Planchet et al., 2014). These results indicate that NO may also be involved in ABA-induced drought stress response. Drought stress stimulates the generation of superoxide radicals (O_2_^-^), H_2_O_2_, and MDA in the leaves of seedlings, thus the growths of Pusa Jagannath and Varuna Indian mustards are inhibited (Sahay et al., 2019). However, ABA and NO markedly promote growth by enhancing the activities of antioxidant enzymes, mediating proline (Pro) metabolism, and maintaining water content (Sahay et al., 2019; **Table 1**). Pontin et al. (2021) also conclude that ABA and NO can promote drought tolerance by improving antioxidant enzyme activities and flavonoid metabolism and maintaining water balance in the leaves of grapevines.

**Table 1:** Overview of the regulatory pathways of ABA-NO interaction under abiotic stresses in plants.

Stress factor	Stress conditions	Plants	Tissues	ABA and NO roles under stress	Regulatory Pathways	Antioxidant enzymes	References
Drought	Withholding irrigation	Grapevine	Leaves	• Reduced water potential, leaf area, leaves dry mass • Increased shoot length	• Antioxidant enzymes • Flavonoids pathway • Water homeostasis • Photosynthesis	APX↑, POD↑, CAT↓	Pontin et al., 2021
	PEG	Indian mustard	Leaves/Roots	• Increased leaf RWC, chlorophyll, carotenoid, protein contents • Increased ABA, NO contents • Reduced MDA, H_2_O_2_, O_2_^-^	• Antioxidant enzymes • AsA-GSH cycle • Proline pathway • Flavonoids pathway • Water homeostasis	In the roots: SOD↑, CAT↑, APX↓, GR↓ In the leaves: SOD↓, CAT↑, APX↑, GR↓	Sahay et al., 2019
	PEG	Medicago	Seedlings	• Promoted NO, Proline accumulations • Reduced germination rate, water content	• Proline pathway • Water homeostasis	–	Planchet et al., 2014
	PEG	Bromeliaceae	Leaves	• Promoted ABA, NO, and H_2_O_2_ accumulations	• Water homeostasis	–	Mioto and Mercier, 2013
	PEG	Wheat	Root tips	• Promoted ABA, H_2_O_2_, NO formations	• ROS	–	Tari et al., 2010
	Withholding irrigation	Tb	Seedlings	• Increased RWC, photosynthetic capacity • Reduced ion leakage, MDA, plant death rate • Promoted H_2_O_2_ and NO formation	• ROS • Antioxidant enzymes • Photosynthesis • Water homeostasis	SOD↑, CAT↑	Lu et al., 2009
	0.4 M Mannitol	Wheat	Seedlings	• Reduced water loss	• Water homeostasis	–	Xing et al., 2004
	Evaporated at room temperature	Wheat	Seedlings	• Promoted ABA formation	• ROS	–	Zhao et al., 2001
Salt	NaCl	Rice	Seedlings	• Promoted ABA, NO formation • Increased RWC, Na^+^/K^+^ ratio, osmotic potential • Reduced H_2_O_2_, O_2_^-^	• Antioxidant enzymes • Polyamines pathway, • Water homeostasis	APX↑, GST↑	Saha et al., 2022
	NaCl	Tomato	Roots	• Reduced root length, MDA • Promoted NO formation	• Antioxidant enzymes • Water homeostasis	APX↑, CAT↑	Santos et al., 2020
	NaCl	Wheat	Leaves	• Promoted ABA and Proline formations • Increased RWC	• Proline pathway • Water homeostasis	–	Ruan et al., 2004
HM	Mo	Winter wheat	Seedlings	• Promoted ABA • Reduced MDA	• Antioxidant enzymes	APX↑, CAT↑, SOD↑, POD↑	Wu et al., 2018
	Pb	Cowpeas	Leaves	• Increased stomatal conductance, leaf area, seed yield • Promoted IAA, CTK, ABA, and GA3 formations	• Hormone crosstalk	–	Sadeghipour, 2017
	Al	Rye and wheat	Roots	• Increased root elongation • Promoted IAA, ABA, and GA formations	• Hormone crosstalk	–	He et al., 2012
High light	500 μmol m^−2^ s^−1^ PPFD	Tall fescue	Seedlings	• Reduce ion leakage, MDA, ROS • Enhanced NO synthesis	• Antioxidant enzymes	APX↑, CAT↑, SOD↑, GR↑	Xu et al., 2013
Low light	40 μmol m^−2^ s^−1^ PPFD	Tall fescue	Seedlings	• Increased plant height, leaf width, tiller number, dry weight • Enhanced photosynthetic capacity • Reduced ion leakage, MDA, ROS	• Antioxidant enzymes • Photosynthesis	APX↑, CAT↑, SOD↑, POD↑	Zhang et al., 2018b
UV-B	3.3 W m^-2^ irradiance	Maize	Seedlings	• Promoted NO, ABA, and H_2_O_2_ formations	• ROS • ABA	–	Tossi et al., 2009
Heat	40 °C for 6 h	Wheat	Seedlings	• Increased plant length, leaf area, plant fresh weight, plant dry weight • Enhanced photosynthetic capacity • Reduced ROS, TBARS • Promoted NO, ABA formations • Promote osmoregulator production	• Antioxidant enzymes • Proline pathway • Water homeostasis • Photosynthesis	APX↑, CAT↑, SOD↑, GR↑	Iqbal et al., 2022
	45 °C in the dark for 2 h	Reed	Seedlings	• Reduced membrane permeability, MDA • Increased relative growth gate • Promoted NO, ABA formations	• ABA	–	Song et al., 2008
Chilling	CA at 12 °C for 3 d; exposed to 4 °C for 5 d	Tomato	Seedlings	• Promoted NO, ABA, and H_2_O_2_ formations	• Photosynthesis	–	Lv et al., 2018
	Pre-cooled at 0 °C for 24h	Peach	Fruits	• Reduced chilling index • Increased firmness, soluble solids content, peel color • Reduced ROS, electrolyte leakage, MDA	• Antioxidant enzymes • AsA-GSH cycle	APX↑, PAD↑, SOD↑, GR↑	Zhang et al., 2019
	4 °C for 3 d	Walnut	Shoot leaves	• Reduced ROS, MDA	• Antioxidant enzymes • AsA-GSH cycle	APX↑, CAT↑, SOD↑, GR↑	Dong et al., 2017
	4 °C for 1 d	Tomato	Seedlings	• Promoted NO, ABA, H_2_O_2_, polyamines, formations	• Polyamines pathway	–	Diao et al., 2017
	-3 °C for 3 h	*Medicago*	Seedlings	• Reduced ion leakage • Increased survival rate	• Antioxidant enzymes • Photosynthesis	APX↑, CAT↑, SOD↑	Guo et al., 2014
	1 °C in the dark for 6 d	Maize	Seedlings	• Increased survival percentage • Reduced ROS, MDA	• ROS • Antioxidant enzymes	CAT↑, SOD↑	Li and Zhang, 2012
Freezing	−15 °C for 1 d	Cl	Leaves	• Reduced stomatal aperture • Promoted NO, ABA, flavonoids, formations	• Antioxidant enzymes • Flavonoids pathway	CAT↑, SOD↑	Peng et al., 2022
Alkali	NaHCO_3_: Na_2_CO_3 = _9:1; 0-150 mM for 12 d	*NT*	Seedlings	• Increased fresh weight, plant height, RWC, degree of succulency, Na^+^/K^+^ ratios • Reduced electrical leakage, ROS, MDA, stomatal aperture • Accumulation of osmoregulators	• Antioxidant enzymes • AsA-GSH cycle • Water homeostasis • Photosynthesis • Stomatal movement	APX↑, CAT↑, SOD↑, GR↑, GST↑	Zhang et al., 2023

ABA, abscisic acid; NO, nitric oxide; AsA-GSH cycle, ascorbate-glutathione cycle; PEG, polyethylene glycol; RWC, relative water content; ROS, reactive oxygen species; MDA, malondialdehyde; H_2_O_2_, hydrogen peroxide; O_2_^-^, superoxide radicals; Tb, Triploid bermudagrass; Mo, molybdenum; Pb, lead; Al, aluminum; HM, Heavy metal; UV-B, Ultraviolet-B; PPFD, photosynthetic photon flux density; CA, cold acclimation; TBARS, thiobarbituric acid reactive substances; Cl, Creeping lilyturf, *NT*, *Nitraria tangutorum*; CAT, catalase; SOD, superoxide dismutase; GR, glutathione reductase; APX, ascorbate peroxidase; POD, peroxidase; GST, glutathione S-transferase; IAA, auxin; CTK, cytokinin; GA, gibberellic acid.

Overall, previous reports exhibit that the involvement of NO in ABA-induced tolerance is linked to H_2_O_2_. At the same time, crosstalk between ABA and NO confers drought tolerance in plants by maintaining water balance, enhancing antioxidant-related enzymes, and promoting flavonoid metabolism. Nevertheless, there are few studies on the role of ABA-NO interaction in drought tolerance at the molecular aspects, this topic needs to be further investigated.

### 2.2 Temperature stress

Temperature stress, including high-temperature stress and low-temperature stress, restricts the growth and development of plants. High temperature induces ABA and NO accumulation in reed plants (Song et al., 2008). In addition, the effects of NO scavenger/inhibitor cPTIO/N^w^-nitro-L-arginine (LNNA) on endogenous ABA content are not significant, however, endogenous NO content and NOS-like activity are decreased by ABA inhibitor Flu. The results suggest that NO production is involved in ABA-induced high-temperature stress tolerance and acts downstream of ABA (Song et al., 2008; **Table 1**). Iqbal et al. (2022) conclude that heat tolerance is enhanced by ABA and 100 µM SNP by improving photosynthesis and antioxidant capacity and up-regulating the expression of antioxidant enzyme-related genes. Applying 100 µM ABA and SNP can alleviate low-temperature-induced growth inhibition of maize seedlings via enhancing antioxidant enzyme activities and reducing H_2_O_2_ and MDA levels (Li and Zhang, 2012; **Tables 1**, **2**). Furthermore, the effects of ABA are blocked by NO scavenger, indicating that ABA enhances chilling tolerance by NO generation. Similarly, Dong et al. (2017) report that ABA and NO significantly enhance antioxidant response in the leaves of walnut shoots under low-temperature stress. Nevertheless, co-treatment with cPTIO and ABA can reverse the effects. The exposure of peach fruits to low temperatures significantly increases endogenous ABA and NO contents, therefore enhancing antioxidant capacities to eliminate excess ROS and preventing over-oxidation of the plasma membrane (Zhang et al., 2018b; **Table 1**). In addition, the effect is inhibited by NO scavenger/inhibitor, suggesting that ABA induces the antioxidant response to confer low-temperature tolerance through NO production.

**Table 2:** Overview of ABA-and NO-mediated genes under abiotic stresses in plants.

Stresses	Plants	Tissues	Genes	Roles of genes	References
Salt	Rice	Roots	*OsMSRA4*, *OsMSRA5*, *OsMSRB1.1*, *OsMSRB3*, *OsMSRB5*	Alleviation of cellular damage	Hsu and Lee, 2018
HM	Winter wheat	Seedlings	*TaSOD*, *TaCAT*, *TaAPX*	Activation of antioxidant enzymes	Wu et al., 2018
Low light	Tall fescue	Seedlings	*POD*, *CnZn-SOD*, *Mn-SOD*, *CAT-A*, *CAT-B*, *APX2*, *APX4*, *FaNOA1*, *CAT-C*, *FaPYR1*, *FaPYL1*	Activation of antioxidant enzymes, Promotion of ABA synthesis	Zhang et al., 2018b
Heat	Wheat	Seedlings	*APX*, *GR*	Activation of antioxidant enzymes	Iqbal et al., 2022
Chilling	Tomato	Seedlings	*CPK27*	Regulation of ROS, NO, and MPK1/2	Lv et al., 2018
	Tomato	Seedlings	*LeNR*, *LeNOS*, *LeADC*, *LeADC1*, *LeODC*, *LeSPDS*, *LeNCED1*	Promotion of ABA and polyamine synthesis	Diao et al., 2017
	*Medicago*	Seedlings	*MfSAMS1*, *PAO*, *CuAO1*, *CuAO2*, *Cu-SOD*, *Zn-SOD*, *CAT1*, *cAPX*, c*pAPX*, *SAMDC1*, *SAMDC2*, *SPDS1*, *SPDS2*, *SPMS*	Promotion of PAs biosynthesis, Activation of antioxidant enzymes	Guo et al., 2014
Freezing	Cl	Leaves	*C4H*, *CHS*, *CHI*, *F3H*, *F3’5’H*, *F3’H*, *FLS*, *LUT5*, *ZEP*, *NCED*, *ABA2*, *AAO3*, *VED*, *NOS, PAO*	Promotion of flavonol synthesis	Peng et al., 2022
Alkali	*NT*	Seedlings	*NtNOA1*, *NtNR2, NtNCED1/3/4/5*, *NtAAO*, *NtSDR, NtPYL2/6*, *NtPP2C*, *NtABF1/3*, *NtSnRK2.2/2.3, NtSOS1, NtNHX1/2/3, NtKEA 3/5, NtKUP4*, *NtKCO*, *NtHAK6/12, NtHKT1*	Inhibition of heavy metal ion uptake, Promotion of ABA and NO synthesis	Zhang et al., 2023

HM, Heavy metal; Cl, Creeping lilyturf; ROS, reactive oxygen species; NO, nitric oxide; PAs, polyamines; MPK1/2, calcium mitogen-activated protein kinases 1/2; NT, Nitraria tangutorum.

Low temperature up-regulates the expression level of the *MfSAMS1* gene (related to S-adenosylmethionine synthetase, the enzyme catalyzing the formation of polyamines and ethylene), manifesting that *MfSAMS1* may be involved in the response to cold stress in *Medicago* (Guo et al., 2014; **Tables 1**, **2**). Additionally, ABA, H_2_O_2_, or NO treatment alone significantly up-regulates *MfSAMS1* expression. And its expression is decreased by ABA biosynthesis inhibitor naproxen, H_2_O_2_ scavenger dimethylthiourea (DMTU), or NO scavenger cPTIO treatment under cold stress, respectively, indicating that ABA, H_2_O_2_, or NO influences *MfSAMS1* expression and they may alleviate cold stress by up-regulating *MfSAMS1* expression. In order to further verify the role of *MfSAMS1* under cold stress, overexpressing *MfSAMS1* transgenic tobacco is obtained (Guo et al., 2014). The overexpression of *MfSAMS1* in tobacco increases the survival and net photosynthetic rate (Pn) as well as decreases ion leakage. The contents of spermidine (Spd) and putrescine (Put) are also improved, following high levels of enzyme activities and gene expression related to polyamine biosynthesis and antioxidant enzyme (Guo et al., 2014). These results reveal that SAMS alleviates cold stress through up-regulating polyamine oxidation. Likewise, the concentrations of NO and H_2_O_2_ are raised by Spd or Spm treatment alone in tomato seedlings under low-temperature stress (Diao et al., 2017). Furthermore, Spd or Spm can enhance the activities of nitrate reductase (NR, EC 1.7.99.4), NOS-like, diamine oxidase (DAO, EC 1.4.3.22), and polyamine oxidase (PAO, EC 1.5.3.11), up-regulating *LeNR* expression, and down-regulating *LeNOS1* expression (Diao et al., 2017). Endogenous NO content is decreased when H_2_O_2_ inhibitor or scavenger is added, while endogenous H_2_O_2_ content is not significantly changed when NO inhibitor or scavenger is added (Diao et al., 2017). The results mentioned above indicate that Spd and Spm enhance the production of NO by the NR pathway depending on H_2_O_2_. SNP treatment increases Put, Spd, and Spm contents and up-regulates polyamine biosynthesis-related gene expression, so it can be concluded that NO can increase the contents of Put and Spd under low-temperature stress. ABA content is increased by Put treatment, following the reduction of electrolyte leakage. However, the effects are reversed by Put synthesis inhibitor D-arginine. The expression of the ABA biosynthesis-related gene is up-regulated by Put treatment, but it is not observably changed by D-Arg, showing that ABA is involved in Put-induced low-temperature tolerance (Diao et al., 2017). These results demonstrate that interaction between NO, ABA, and H_2_O_2_ can improve the PAs-induced cold tolerance by up-regulating the expression of polyamine and ABA biosynthesis-related gene expression (**Tables 1**, **2**). In *CPK27*-silenced (related to calcium-dependent protein kinases) tomatoes, photosynthetic and antioxidant capacity, ABA, NO, and H_2_O_2_ contents, and the activities of nicotinamide adenine dinucleotide phosphate (NADPH) oxidase, NR, and mitogen-activated protein kinases1/2 (MPK1/2) are decreased under cold stress (Lv et al., 2018). However, the levels of NO, H_2_O_2_, and MPK1/2 are increased when ABA is added during the process. To study the relationship among ABA, NO, H_2_O_2_, and MPK1/2, the mutants are obtained. The results indicate that low level of ABA is detected in the silence of gene related to H_2_O_2_-synthesis *RBOH1* (NADPH-dependent respiratory burst oxidase homolog), *MPK1/2*, or *NR* mutant, separately. Furthermore, low levels of *CPK27*, NO, and MPK1/2 are found in the deficiency of ABA mutant *notabilis* (Lv et al., 2018; **Tables 1**, **2**). The results reflect that ABA is involved in the upregulation of *CPK27* expression-induced low-temperature tolerance by increasing NO, H_2_O_2_, and MPK1/2 levels. After cold-acclimated treatment, creeping lilyturf leaves have higher water retention capacity, photosynthetic efficiency, and antioxidant enzyme activity (Peng et al., 2022; **Tables 1**, **2**). The authors use transcriptomics and metabolomics to find that genes related to cellular processes, environmental information processing, genetic information processing, metabolism, and organismal systems are considerably enriched, with 4,620 upregulation and 5,824 downregulation (Peng et al., 2022). The authors identify that the contents of 10 flavonoids increased significantly under low-temperature stress, following regulation of the expression of flavonol biosynthesis pathway genes (Peng et al., 2022). Likewise, the contents of intermediate products and genes associated with the ABA biosynthesis pathway are significantly regulated, following high ABA concentration and low stomatal aperture. The sequence of ABA signaling is sequentially from PYR1/PYL, PP2C, SnRK2 to ABF. In addition, NO content is increased, with *NOS* up-regulation and *PAO* down-regulation (Peng et al., 2022; **Table 2**). The intermediates of the NO biosynthesis pathway are increased. These results exhibit that the interaction between ABA, NO, and flavonoids can improve low-temperature tolerance via reducing stomatal aperture.

Similar to drought tolerance, ABA-NO interaction can improve tolerance through enhancing antioxidant response and photosynthetic capacity, up-regulating *MfSAMS1* and *CPK27* expression, promoting polyamine and flavonoid biosynthesis, and accelerating stomatal closure.

### 2.3 Salt

Excessive salt is one of the most important factors restricting crop growth (Elsiddig et al., 2022). Santos et al. (2020) found that endogenous NO content is increased in the roots of wild-type (WT) tomato but reduced in the roots of ABA-insensitive mutant *sitiens* under salt stress. The concentration of NO is reduced in WT under salt stress when NO scavenger/inhibitor is added separately, but there is no significant change in *sitiens* mutant (Santos et al., 2020). Meanwhile, SNP treatment can enhance antioxidant enzyme activity in *sitiens* mutant, but WT type is not detected, implying that ABA-mediated NO improves sodium chloride (NaCl) tolerance through enhancing antioxidant enzyme activities (**Table 1**). Ruan et al. (2004) showed that 0.1 mM SNP can promote RWC in the leaves of wheat seedlings under 150 mM NaCl stress. Endogenous ABA content is increased by SNP treatment, but it also can be arrested by NO scavengers (Ruan et al., 2004). Simultaneously, ABA and NO increase Pro content, however NO scavengers suppress it. The results suggest that ABA increases Pro level dependent on NO production under NaCl stress (**Table 1**). The application of ABA and NO can improve the growth of rice by maintaining water homeostasis, sodium ion/potassium ion (Na^+^/K^+^) ratio, elevating antioxidant enzyme activities and antioxidant contents under 200 mM NaCl stress (Saha et al., 2022; **Table 1**). Hsu and Lee, 2018 reveal that methionine sulfoxide reductase (MSR; catalyzed the reduction of methionine sulfoxide to methionine residues and alleviated ROS damage to proteins)-related gene expression is up-regulated in rice roots under salt stress, but when 0.3 mM Flu or 300 μM cPTIO is added, the effect is reversed (Hsu and Lee, 2018; **Table 2**). In this process, NO content is decreased by Flu treatment (Hsu and Lee, 2018), implying that ABA-induced NO responds to salt stress through the regulation of MSR-related genes.

Overall, ABA-induced NO enhances the tolerance against salt stress by maintaining water homeostasis, increasing antioxidant enzyme activity and antioxidant contents, and regulating MSR-related gene expression. There are fewer studies in the molecular direction, and our future research should pay attention to this aspect.

### 2.4 Heavy metal

Excessive heavy metal ions can be absorbed by plants, thus causing damage to the plant cell membrane system (Yu et al., 2023). Wu et al. (2018) found that ABA synthesis is activated by aldehyde oxidase involved in the ABA biosynthesis pathway under molybdenum (Mo) condition. However, NO scavenger cPTIO can constrain ABA synthesis, following the reduction of antioxidant enzyme activities and associated gene expression. The results show that ABA-dependent NO improves Mo tolerance by activation of the antioxidant systems. Lead (Pb) results in a reduction of stomatal conductance, leaf area, and seed yield of cowpeas (Sadeghipour, 2017). Besides, the levels of auxin (IAA), cytokinin (CTK), and gibberellic acid (GA) are reduced and ABA is increased. However, 0.5 mM SNP can reverse the above effects. These results show that NO modulates ABA to inhibit Pb uptake, thus enhancing Pb tolerance. SNP treatment promotes root elongation as well as ABA and IAA generations in the root apices of rye and wheat under aluminum (Al) stress (He et al., 2012). The effect is reversed by NO scavenger. At present, there are relatively few studies on the interaction between ABA and NO in heavy metal stress tolerance, thus we still need to focus on heavy metal stress.

### 2.5 Light

Generally, light stress includes high-light and low-light stresses (Xu et al., 2013; Zhang et al., 2018b). High-light stress [500 μmol m^−2^ s^−1^ photosynthetic photon flux density (PPFD); the control is set to 100 μmol m^−2^ s^−1^ PPFD] results in an increase in ion leakage and an accumulation of MDA in the leaves of tall fescue seedlings (Xu et al., 2013). These negative effects are alleviated by ABA, SNP, or SNP+Flu treatment and can be worsened by cPTIO, Flu, ABA+cPTIO, LNNA, or LNNA+ABA treatment. Furthermore, exogenous ABA increases NOS activity resulting in NO production and antioxidant enzyme activation, implying that ABA alleviates high-light stress depending on NO via enhancing antioxidant capacity (**Table 1**). Zhang et al. (2018b) found that NO content and NOS-like activity in tall fescue seedlings are increased by exogenous ABA and are reduced by Flu under low-light stress (40 μmol m^−2^ s^−1^ PPFD; the control was set to 200 μmol m^−2^ s^−1^ PPFD). Meanwhile, the expression of the NO biosynthesis-related gene is up-regulated by ABA and is descended by Flu. The authors further found that exogenous ABA can raise photosynthetic capacity and antioxidant enzyme activity and up-regulate antioxidant enzyme-related gene expression (Zhang et al., 2018b; **Tables 1**, **2**). To sum up, ABA-activated NOS-like signaling induces NO production and enhances light tolerance through increasing antioxidant and photosynthetic capacity and regulating their gene expression.

### 2.6 UV-B

When the environment tends to continue to deteriorate, more and more ultraviolet irradiation is not absorbed by the ozone layer, resulting in plant exposure to high levels of UV-B radiation, which reduces crop yields. Ion leakage is increased by UV-B in maize seedlings, and this negative effect is alleviated by using exogenous ABA (Tossi et al., 2009). Moreover, the concentration of H_2_O_2_ is increased in maize seedlings of WT, and *viviparous 14* (*vp14*), a mutant defective in ABA synthesis, only slightly changed. NO is detected in the WT but not in the *vp14* mutant (Tossi et al., 2009). In addition, when 100 µM ABA is supplied, NO production is restored in the *vp14* mutant. Catalase (CAT, EC 1.11.1.6, as H_2_O_2_-degrading enzyme), H_2_O_2_ inhibitor diphenylene iodonium (DPI), and the NO inhibitor N^w^-nitro-L-arginine methyl ester (LNAME) partially inhibit UV-B-induced NO accumulation, indicating that H_2_O_2_ and NOS-like activity play an important role in plant response to UV-B (Tossi et al., 2009). The results as mentioned show that UV-B improves ABA accumulation, which activates NADPH oxidase and H_2_O_2_ production, and that a NOS-like-dependent mechanism increases NO production, suggesting that ABA alleviates UV-B by increasing H_2_O_2_ generation and NO production-dependent on NOS-like (**Table 1**). Thus, detailed molecular insights into the underpinning mechanisms remain to be established, for example, UV-B irradiation stress regulates the change of endogenous ABA and NO levels.

### 2.7 Alkali

Plants are subject to alkali stress resulting in growth inhibition. For instance, when *Nitraria tangutorum* seedlings are exposed to alkali stress, plant height, fresh weight, and RWC are reduced (Zhang et al., 2023). Nevertheless, exogenous ABA and NO can promote plant growth of *Nitraria tangutorum* seedlings. The authors further found that exogenous ABA and NO can enhance antioxidant capacity and photosynthetic capacity, regulate transcript levels of ion transporter genes and the biosynthesis of NO and ABA genes, and reduce the stomatal aperture (Zhang et al., 2023). It can be concluded that crosstalk between ABA and NO can improve alkali tolerance by the above pathways.

## 3 Regulatory pathways of NO-ABA interaction alleviating abiotic stress in plants

### 3.1 Reactive oxygen species

Low concentrations of ROS can act as signaling molecules to be involved in ABA and NO interaction under stresses. For example, ROS, ABA, and NO are induced by osmotic stress in drought-sensitive wheat cultivar, implying that there is an interaction among ROS, ABA, and NO under drought stress (Tari et al., 2010; **Table 1**). ABA-induced osmotic tolerance is weakened by NO scavenger/inhibitor and ROS scavenger in the root tips of wheat seedlings (Zhao et al., 2001). Lu et al. (2009) point out that NO and H_2_O_2_ are generated by exogenous ABA in triploid bermudagrass under drought stress. Moreover, ABA-induced oxidative response is inhibited by H_2_O_2_ scavenger/inhibitor and NO scavenger/inhibitor. However, H_2_O_2_ scavenger/inhibitor reduces NO-induced oxidative tolerance. Under UV-B stress, NO and H_2_O_2_ are lower in ABA synthesis-deficient mutant *vp14* compared to WT (Tossi et al., 2009). In addition, NO synthesis is inhibited by H_2_O_2_ inhibitors during the process. The results imply that interaction between ABA and NO is linked by ROS under abiotic stress.

### 3.2 Antioxidant enzymes

**Figure 1:**
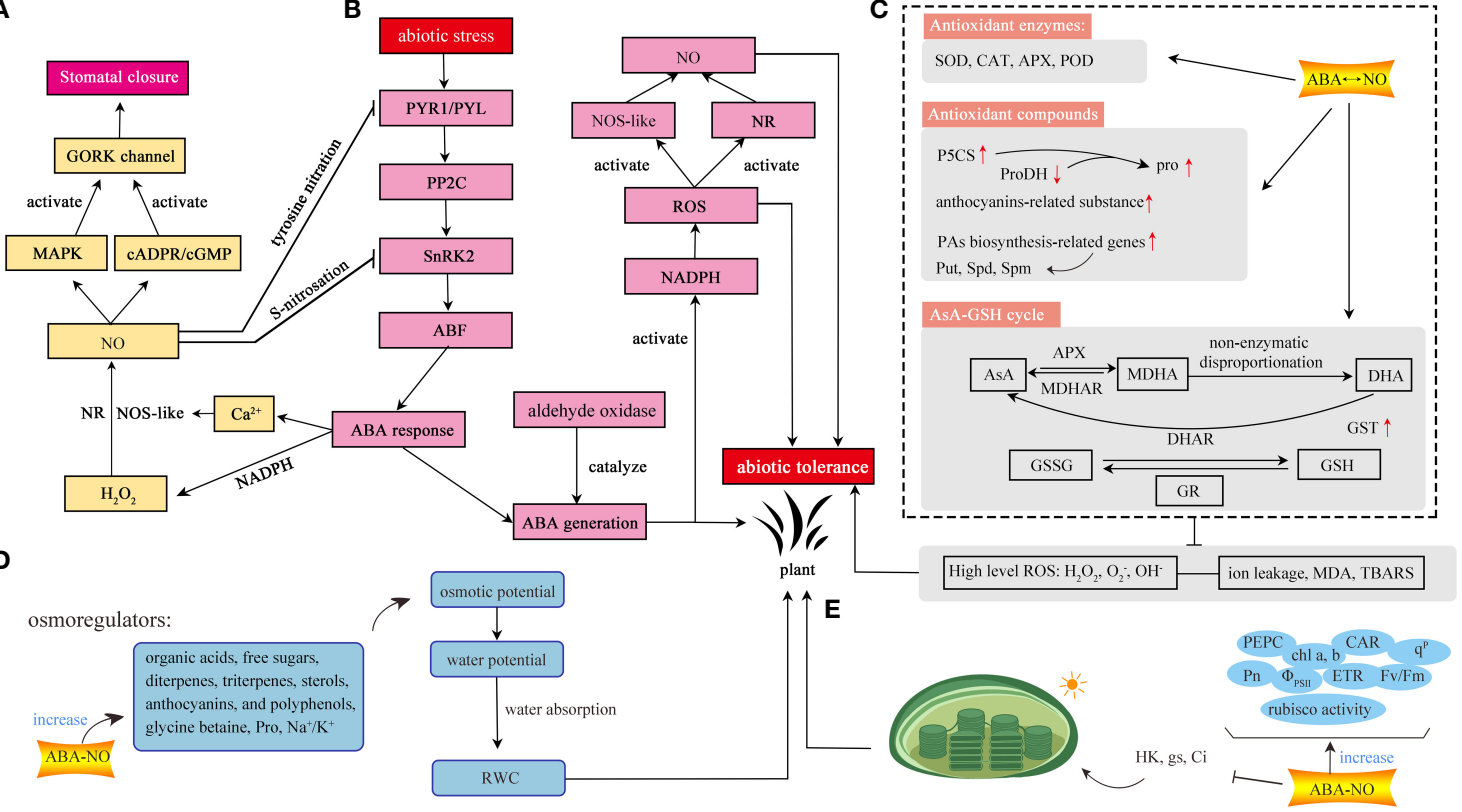
The regulatory mechanism of ABA-NO interaction under abiotic stress in plants. **(A)** Stomatal movement. **(B)** Regulatory pathways among ABA, NO, ROS. **(C)** Antioxidant systems. Plants scavenge excess ROS through three pathways, including antioxidant enzymes, antioxidants, and AsA-GSH cycle. **(D)** Water balance. **(E)** Photosynthesis. The interaction between ABA and NO can mediate photosynthesis to enhance environmental tolerance. GORK, outward-rectifying K^+^; MAPK, mitogen-activated protein kinase; cGMP, 3’,5’-cyclic guanosine monophosphate; NO, nitric oxide; ABA, abscisic acid; NR, nitrate reductase; NOS-like, NO synthase-like; H_2_O_2_, hydrogen peroxide; Ca^2+^, calcium ion; NADPH, nicotinamide adenine dinucleotide phosphate; PYR/PYL, pyrabactin resistance/PYR1-like; PP2C, type 2C protein-phosphatase; SnRK2, sucrose non-fermenting 1-related protein kinase subfamily 2; ABF, ABA-responsive element binding factors; SOD, superoxide dismutase; CAT, catalase; APX, ascorbate peroxidase; POD, peroxidase; P5CS, pyrroline-5-carboxylate synthetase; ProDH, recombinant proline dehydrogenase; PAs, polyamines; Put, putrescine; Spd, spermidine; Spm, spermine; AsA, ascorbic acid; MDHAR, monodehydroascorbate reductase; MDHA, monodehydroascorbate; DHAR; dehydroascorbate reductase; DHA; dehydroascorbate; GST, glutathione S-transferase; GSH, glutathione; GR, glutathione reductase; GSSG, oxidized glutathione; O_2_^-^, superoxide radicals; H_2_O_2_, OH^-^, hydroxyl radicals;hydrogen peroxide; MDA, malondialdehyde; TBARS, thiobarbituric acid reactive substances; hexose kinase; gs, stomatal conductance; Ci, intercellular CO_2_ concentration; PEPC, phosphoenolpyruvate carboxylase; Pn, net photosynthetic rate; chl a, b, chlorophyll a, b; Φ_PSII_, actual photochemical efficiency of PSII; CAR, total carotenoids; ETR, apparent electron transport rate; F_v_/F_m_, the maximum quantum yield of PSII photochemistry; q^P^, photochemical quenching; Pro, proline; RWC, relative water content; Na^+^/K^+^, sodium ion/potassium ion.

Drought-induced oxidative stress results in an elevation of ion leakage, MDA, and H_2_O_2_ in triploid bermudagrass (Lu et al., 2009). To avoid high levels of drought-induced ROS damage to plant cells, plants depend on the enzymatic antioxidant system to scavenge excess ROS. Consequently, the activities of superoxide dismutase (SOD, EC 1.15.1.1), CAT, ascorbate peroxidase (APX, EC 1.11.1.11), and peroxidase (POD, EC 1.11.1.7) are enhanced by NO and ABA to scavenge excess ROS. In addition, ABA and NO-induced antioxidant enzyme activities are inhibited by NO scavenger/inhibitor and H_2_O_2_ scavenger/inhibitor (Lu et al., 2009; **Table 1**; **Figure 1**). The above results show that ABA activates ROS signaling resulting in NO accumulation to enhance antioxidant enzyme activities under drought stress. Drought-induced high levels of O_2_^-^, H_2_O_2_, and MDA are reversed by ABA and SNP alone or co-treatment in Pusa Jagannath and Varuna types of Indian mustard (Sahay et al., 2019; **Figure 1**). The activities of SOD, CAT, and APX in the roots and leaves of Pusa Jagannath are enhanced by NO and ABA during the process. When ABA and SNP are applied, APX and POD activities are elevated in the leaves of grapevines under drought stress (Pontin et al., 2021). Under salt stress, NO is not generated in the root of ABA-insensitive mutant *sitiens*. However, salt resistance is elevated by the activities of APX and CAT compared to WT-type tomato when NO is added (Santos et al, 2020). The results can be seen that NO acts cooperatively in ABA-induced antioxidant enzymes under salt tolerance. Using 200 mM NaCl-induced salt stress triggers the accumulation of H_2_O_2_ and O_2_^-^ in rice, but when SNP and ABA are applied, these negative effects are reversed, following high levels of APX and glutathione S-transferase (GST, EC 2.5.1.18; Saha et al., 2022). Wu et al. (2018) found that the concentrations of O_2_^-^ and MDA are increased in wheat seedlings under Mo condition, but these adverse effects can be relieved by exogenous ABA and SNP. During the process, ABA and SNP also enhance the activities of antioxidant enzymes, including SOD, CAT, POD, and APX (Wu et al., 2018). Nevertheless, cPTIO also can block ABA biosynthesis and antioxidant enzyme activities (Wu et al., 2018; **Figure 1C**). Under high-light stress, ion leakage, MDA, H_2_O_2_, and O_2_^-^ levels increase significantly in the leaves of tall fescue seedlings, whereas ABA and SNP suppress these negative effects (Xu et al., 2013). However, ABA-induced antioxidant responses are inhibited by NO scavenger/inhibitor (Xu et al., 2013; **Figure 1C**). Furthermore, ABA stimulates NO accumulation via the NOS pathway, following the high levels of SOD, CAT, APX, and glutathione reductase (GR, EC 1.8.1.7) (Xu et al., 2013). Likewise, ABA-dependent on NO increases significantly the activities of POD, SOD, CAT, and APX, thus decreasing remarkably the levels of ion leakage, MDA, H_2_O_2_, and O_2_^-^ contents in tall fescue seedlingsunder low-light stress (Zhang et al., 2018b; **Figure 1C**). Iqbal et al. (2022) found that the activities of SOD, CAT, APX, and GR are enhanced by ABA and NO to decrease TBARS and H_2_O_2_ productions in wheat under heat stress. ABA and NO prevent ROS accumulation and MDA production against low-temperature stress, accompanied by an increase in SOD and CAT activities (Li and Zhang, 2012; **Figure 1C**). The levels of SOD, CAT, APX, and GR are increased by ABA and NO caused by decreasing the contents of electrolyte leakage, MDA, H_2_O_2_, O_2_^-^, and OH^–^ in the leaves of walnut shoots under chilling stress (Dong et al., 2017). Furthermore, NO scavenger cPTIO and ABA inhibitor Flu can inhibit these effects. Low-temperature stress triggers the increase of electrolyte leakage, MDA, and ROS, which lead to lipid peroxidation in peach fruit. Nevertheless, the adverse effect is reversed by ABA and NO through enhancing the activities of SOD, POD, APX, GR, monodehydroascorbate reductase (MDHAR), and dehydroascorbate reductase (DHAR, EC 1.8.5.1) (Zhang et al., 2019; **Figure 1C**). Similarly, Zhang et al. (2023) suggest that ABA and NO can eliminate ROS, MDA, and electrical leakage to improve alkali tolerance by enhancing the levels of GR, CAT, SOD, GST, and APX in *Nitraria tangutorum* seedlings. Together, all these results show that the crosstalk between ABA and NO has an essential role in the activation of antioxidant enzymes response to abiotic stress.

### 3.3 Ascorbate-glutathione cycle

The ascorbate-glutathione (AsA-GSH) cycle is a multistep enzymatic catalytic system for ROS elimination in plant tissues (Sahay et al., 2019; Zhang et al., 2019; Xu et al., 2023; Zhang et al., 2023). Four enzymes cooperatively burst ROS to maintain cellular redox balance, including, monodehydroascorbate reductase (MDAR, EC 1.6.5.4), DHAR, GR, and APX. The application of ABA and NO not only improves ascorbic acid (AsA) content but also elevates APX activity in the leaves of Indian mustard exposed to drought stress (Sahay et al., 2019; **Figure 1C**). Alkali stress-induced levels of reduced glutathione (GSH), GR, and GST are increased by ABA and NO, following the increments of the reduced glutathione/oxidized glutathione (GSH/GSSG) ratio in *Nitraria tangutorum* seedlings (Zhang et al., 2023). Under chilling resistance, the activities of APX, GR, and MDAR are promoted by ABA and NO in peach fruits (Zhang et al., 2019). ABA and NO also induce AsA and GST generations as well as high AsA/DHA and GSH/GSSG ratios, but these effects are inhibited by ABA+cPTIO treatment. Similarly, the levels of AsA, GSH, APX, and GR are raised by ABA and NO in the leaves of walnut shoots under low-temperature stress (Dong et al., 2017; **Figure 1C**). In addition, these effects are reversed by ABA+cPTIO treatment. The results imply that the ABA-induced AsA-GSH cycle exists NO-dependent mechanism.

### 3.4 Proline

Pro is an antioxidant compound that is considered a ROS scavenger (Pontin et al., 2021). Under salt stress, both ABA and NO improve the activity of pyrroline-5-carboxylate synthetase (P5CS, a Pro synthesis enzyme) and inhibit the activity of recombinant proline dehydrogenase (ProDH, a Pro degradation enzyme) in the leaves of wheat seedlings, indicating that Pro participates in the interaction between ABA and NO-induced salt tolerance (Ruan et al., 2004; **Figure 1C**). Sahay et al. (2019) report that ABA and NO increase the content of Pro in the leaves of Pusa Jagannath type Indian mustard under drought stress. Heat stress-induced Pro in wheat is raised by ABA and SNP treatments (Iqbal et al., 2022). When NO scavenger cPTIO is added, the Pro content induced by ABA is reduced. Interestingly, the Pro concentration of *Medicago* seedlings is reduced by NO under drought stress (Planchet et al., 2014). Similarly, the concentration of Pro in grapevines is also reduced by ABA or NO (Pontin et al., 2021). In short, the above results show that Pro may be modulated by ABA-NO interaction under environmental stresses. However, there is an urgent need for further explorations to understand the potential mechanisms. For example, the regulation pathway of Pro content by the interaction between ABA and NO.

### 3.5 Flavonoids

PEG-induced total flavonoid accumulation is reduced by ABA and NO (Sahay et al., 2019), implying that ABA and NO are involved in drought tolerance through flavonoid metabolism (**Table 1**). However, Pontin et al. (2021) show that ABA and NO can improve anthocyanins-related substance contents such as cyanidin 3-O-glucoside, petunidin 3-O-glucoside, malvidin 3-O-glucoside, peonidin 3-O-p-coumaroylglucoside, malvidin 3-O-p-coumaroylglucoside, revealing that ABA and NO can alleviate drought stress by the flavonoid accumulation pathway (**Table 1**; **Figure 1C**). Peng et al. (2022) identify 10 individual flavonoids (Cinnarnoyl-CoA, P-Cinnarnoyl-CoA, Naringenin chalcone, Naringenin, Dihydrokaempferol, Dihydroquercetin, Dihydromyricetin, Kaempferol, Quercetin, Myricetin) and flavonol biosynthesis pathway-related genes under freezing stress by using transcriptomics and metabolomics. Meanwhile, ABA biosynthesis-related genes and intermediates (β-Carotene, β-Cryptoxanthin, Zeaxanthin, Antheraxanthin, Violaxanthin, Neoxanthin, 9’-cis-Violaxanthin, 9’-cis-Neoxanthin, Xanthoxin, Abscisic aldehyde, Xanthoxic acid, and Abscisic alcohol) are significantly up-regulated as well as ABA production and low stomatal aperture. The expression level of *NOS* is also up-regulated, following the production of NO, arginine, and N_ω_-Hydroxy-arginine, implying that ABA-NO interaction relied on flavonoid metabolism to improve low-temperature tolerance (**Table 1**). In general, the detailed response mechanisms of flavonoids by crosstalk between ABA and NO remain to be understood.

### 3.6 Polyamines

PAs are considered antioxidant compounds, including Put, Spd, and Spm in plants. ABA and SNP treatments can increase the content of total PAs in rice seedlings, implying that ABA and NO can respond to salt stress through PAs pathway (Saha et al., 2022; **Table 1**; **Figure 1C**). Under cold stress, the expression level of *MfSAMS1* in *Medicago* is promoted by ABA, H_2_O_2_, or NO, following the productions of Put, Spd, and Spm (Guo et al., 2014). Simultaneously, the above results are suppressed by ABA inhibitor naproxen, H_2_O_2_ scavenger DMTU, and NO scavenger cPTIO (Guo et al., 2014). The results suggest that the interaction between ABA and NO upregulates the expression of key gene for PAs to promote PAs generation under stressful conditions (Guo et al., 2014; **Table 1**; **Figure 1C**). Diao et al. (2017) reveal that the concentrations of Put, Spd, and Spm are increased by SNP treatment in tomato seedlings, following up-regulation of PAs biosynthesis-related gene expression. Moreover, when Spd and Spm are applied, NO and H_2_O_2_ contents are increased as well as *LeNR* upregulation (Diao et al., 2017; **Table 1**). Put treatment can improve ABA concentration and the ABA biosynthesis-related gene is also up-regulated, showing that PAs depend on NO, ABA, and H_2_O_2_ to improve cold tolerance. However, the specific mechanisms by which crosstalk between ABA and NO can affect endogenous PAs accumulation are still not clear and need further study.

### 3.7 Osmotic adjustment

Abiotic stresses usually induce water tolerance and accumulation for various organic and inorganic compounds contributing to osmotic adjustment in plants (Sahay et al., 2019; Pontin et al., 2021). ABA treatment is able to block the RWC reduction and promote the production of NO and H_2_O_2_ in triploid bermudagrass under drought stress, showing that ABA maintains water balance through NO and H_2_O_2_ (Lu et al., 2009; **Table 1**; **Figure 1D**). PEG induces the production of ABA, NO, and H_2_O_2_, following the low water content in *Guzmania monostachia* (Mioto and Mercier, 2013; **Figure 1D**). Planchet et al. (2014) observe that PEG promotes NO production as well as low water content in *Medicago* seedlings. The authors further found that cPTIO increases the water content by osmoregulators accumulation, such as amino acid, glutamate, and Pro (Planchet et al., 2014). In addition, NO is induced by ABA during the process. ABA and NO improve leaf RWC by Pro generation in drought-tolerance of Indian mustard seedlings (Sahay et al., 2019). Under drought stress, the water potential (Ψ_w_) of grapevine seedlings is significantly reduced, which is reversed by ABA and NO (Pontin et al., 2021; **Table 1**; **Figure 1D**). Meanwhile, ABA and NO stimulate the production of organic acids, free sugars, diterpenes, triterpenes, sterols, anthocyanins, and polyphenols. The ratio of leaf water loss is reduced with the accumulation of ABA in wheat seedlings under mannitol-induced osmotic stress (Xing et al., 2004). In this process, when NO inhibitor LNAME or LNNA is applied, ABA content is decreased and water loss is elevated. The results suggest that ABA-induced NO promotes osmosis tolerance by maintaining water balance. ABA and NO can increase osmotic potential by promoting the accumulation of gamma aminobutyric acid, glycine betaine, alanine, and sucrose as well as Na^+^/K^+^ ratio, thus RWC enhancement in rice (Saha et al., 2022; **Table 1**). Similarly, ABA and NO decrease leaf water and stomatal aperture through improving K^+^, betaine, soluble protein, sugar, flavonoid, and Pro contents as well as reducing Na^+^ content under alkali stress (Zhang et al., 2023). Heat tolerance-induced osmolyte contents are raised by ABA and NO, including Pro, glycine betaine, trehalose, and soluble sugar in the leaves of wheat seedlings (Iqbal et al., 2022). However, ABA-induced osmolytes are inhibited by NO scavenger cPTIO. Although the studies described above suggest that crosstalk between ABA and NO can maintain water balance by regulating osmolytes, the mechanisms underlying the regulation of osmolytes by interaction between ABA and NO need to be further explored.

### 3.8 Photosynthesis

ABA, NO, and H_2_O_2_ can enhance phosphoenolpyruvate carboxylase (PEPC, an enzyme involved in photosynthesis) activity under environmental stress, revealing that they play a vital function in photosynthesis under abiotic tolerance (Lu et al., 2009; Mioto and Mercier, 2013). Under osmotic stress, chlorophyll (chl) content is elevated by ABA and NO (Pontin et al., 2021). This is the result of ABA and NO increasing water potential and thus promoting stomatal closure. ABA improves the low-light tolerance of tall fescue seedlings via enhancing photosynthesis, such as chl a, chl b, total carotenoids (CAR) contents, Pn, the maximum quantum yield of PSII photochemistry (F_v_/F_m_), actual photochemical efficiency of PSII (Φ_PSII_), photochemical quenching (q^P^), apparent electron transport rate (ETR), consequently, decreasing intercellular CO_2_ concentration (Ci) (Zhang et al., 2018b; **Figure 1E**). More importantly, NO and H_2_O_2_ are generated by ABA treatment during the process. The above results imply that NO and H_2_O_2_ contribute to ABA-activated photosynthesis. Iqbal et al. (2022) also report that heat-induced decrease chl content, Pn, stomatal conductance (gs), Ci, F_v_/F_m_, and rubisco activity, but ABA and NO reverse the effects, showing that ABA and NO can improve the heat-tolerance by enhancing photosynthetic capacity in wheat. However, the effects are worsened by ABA inhibitor Flu or NO scavenger cPTIO. Under alkali stress, photosynthetic capacity is reduced in *Nitraria tangutorum* seedlings (Zhang et al., 2023). However, ABA and NO increase in the levels of Chl a, Chl b, CAR contents, F_v_/F_m_, ETR, non-photochemical quenching (NPQ), Φ_PSII_, Pn and reduce levels of q^P^ and Ci, therefore enhancement of photosynthesis. To summarize, the interaction between ABA and NO increases photosynthetic capacity by altering photosynthetic indexes, however, the relevant mechanisms are missing.

### 3.9 Stomatal movement

As a first line of defense, stomatal closure plays a vital in stress. It has been reported that ABA promotes stomatal closure in many plant species. Furthermore, previous studies demonstrate that ABA-induced stomatal closure is reversed by NO scavengers (Garcia-Mata and Lamattina, 2002; Neill et al., 2002b), thus NO is also involved in stomatal movement. However, this is a complex process in guard cells, with many signaling cascade involvement. For example, H_2_O_2_-induced stomatal closure is blocked by NO scavengers in *Arabidopsis* (Bright et al., 2006). Moreover, the functions of ABA and H_2_O_2_ in triggering stomatal closure are deficient in NO-deficient double mutant *nia1nia2* in *Arabidopsis*. Interestingly, in NADPH oxidase deficient double mutant *atrbohD/F*, ABA-induced stomatal closure is inhibited with diminishing of NO accumulation (Bright et al., 2006). These results make it clear that ABA mediates H_2_O_2_-trigger NO to regulate stomatal closure. NO biosynthesis is enhanced by ABA thereby activating cADPR and cGMP (3’,5’-cyclic guanosine monophosphate) leading to stomatal closure in pea guard cells (Neill et al., 2002a; **Figure 1A**). The stomatal movement induced by ABA and calcium ions (Ca^2+^) is inhibited by BAPTA-AM (Ca^2+^ chelating agent) in broad bean guard cells (Garcia-Mata and Lamattina, 2007). However, NO-induced stomatal movement is not significantly inhibited during this process. In addition, Ca^2+^ depends on the enhancement of NOS-like activity to increase NO synthesis. ABA can activate the outward-rectifying K^+^ (GORK) channel causing stomatal closure in guard cells (Desikan et al., 2004; Xie et al., 2014). This is a result of ABA-induced H_2_O_2_ production, which is then involved in RbohF-dependent ROS and NR making NO accumulation. On the contrary, ABA signaling can be regulated negatively by NO through S-nitrosation that inhibits open stomatal 1 (OST1)/sucrose nonfermentable 1 (SNF1)-associated protein kinase 2.6 (SnRK2.6) in *Arabidopsis* guard cells (Wang et al., 2015; **Figure 1A**). Under alkali stress, crosstalk between ABA and NO can be reduced stomatal aperture by water regulation (Zhang et al., 2023). Gayatri et al. (2013) report that a signaling cascade under drought: ABA is the first accumulated; then RBOHD and RBOHF (respiratory burst oxidase homologs D and F) are activated leading to an increase in ROS; NO is generated through the activation of NR; mitogen-activated protein kinase (MAPK) signaling is activated by NO eventually resulting in stomatal closure (**Figure 1A**). In short, NO is essential for ABA-induced stomatal movement and requires the involvement of multiple signaling molecules. Nevertheless, we need to concentrate on the mechanisms by which crosstalk between ABA and NO regulates stomatal movement under more abiotic stresses, especially signaling cascades.

### 3.10 Post−translational modifications

S-nitrosylated proteins are detected in *Arabidopsis*, tobacco, and maize under drought or salt stress (Wawer et al., 2010; Mengel et al., 2013; Santisree et al., 2015), implying NO contributes to abiotic tolerance by mediating PTMs. Moreover, the effects of ABA on stomatal movement, growth inhibition, and seed dormancy are modulated by PTMs. For example, NO inhibits ABA-induced stomatal closure by activating S-nitrosation of SnRK2.6/OST1 at the cysteine137 site in *Arabidopsis* guard cells (Wang et al., 2015). NO reverses the effects of ABA-induced growth inhibition and seed dormancy of *Arabidopsis* via generating S-nitrosation of abscisic acid insensitive 5 (ABI5, the basic leucine zipper transcriptional factor, involved in the ABA synthesis) at cysteine153 (Albertos et al., 2015). Similarly, ABA is degraded by the tyrosine nitration of PYR/PYL/RCAR (ABA receptors) at tyrosine23 and tyrosine58 in *Arabidopsis* (Castillo et al., 2015). More importantly, NO drives S-nitrosation of APX1 (ascorbate peroxidase1) at cysteine32 to enhance ROS elimination, therefore improving abiotic tolerance in *Arabidopsis* (Yang et al., 2015). The above results imply that NO-mediated PTMs regulate the protein structure and function of ABA and antioxidant enzymes in response to abiotic stress. Additionally, the mechanism of linkage between ABA and NO is PTMs under abiotic stress. In fact, research that ABA-modulated abiotic tolerance involving S-nitrosation and tyrosine nitration is very poorly known. Therefore, we should focus on developing these areas of NO-mediated transcriptomics, proteomics, and PTMs.

## 4 Regulation of gene expression levels by ABA-NO interaction under environmental stresses

**Figure 2:**
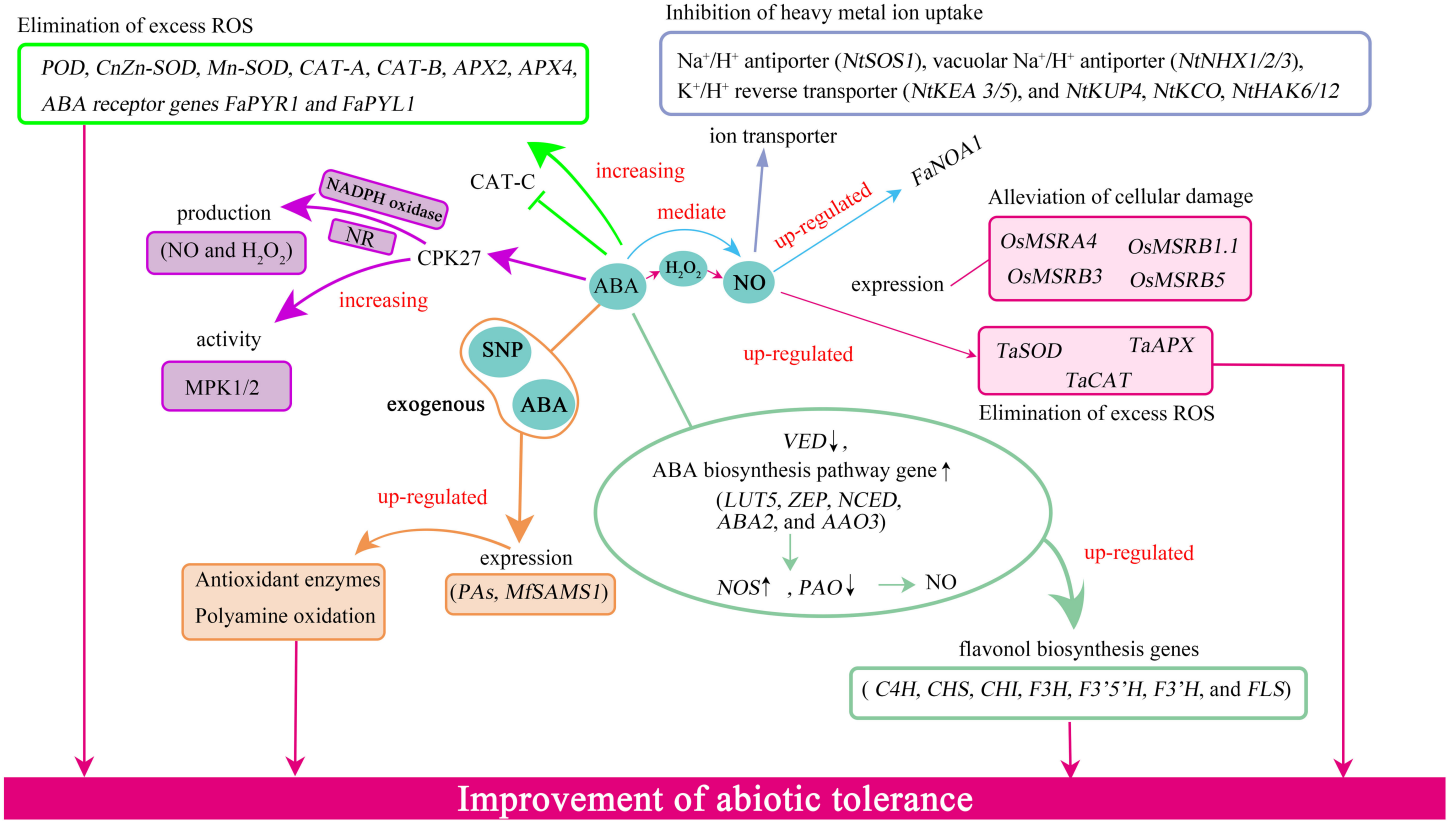
The interaction between ABA and NO regulated genes in plants under environmental stresses. ABA increased the expression levels of *POD*, *CnZn-SOD*, *Mn-SOD*, *CAT-A*, *CAT-B*, *APX2*, *APX4*, *FaNOA1*, *FaPYR1*, and *FaPYL1* as well as NO and H_2_O_2_ production and NADPH oxidase, NR, and MPK 1/2 activities. ABA also up-regulated the expression of flavonol biosynthesis pathways- and ABA biosynthesis-related genes, down-regulated *VED*, reduced PAO activity, enhanced NOS-like activity as well as NO production. The interaction among ABA, NO, and H_2_O_2_ could up-regulate the expression of PAs-related genes as well as *MfSAMS1*. ABA-mediate NO up-regulated the expression of genes related to MSR and antioxidant enzymes. ABA, abscisic acid; NO, nitric oxide; SNP, sodium nitroprusside dihydrate; H_2_O_2,_ hydrogen peroxide; PAs, polyamines; NADPH, nicotinamide adenine dinucleotide phosphate; NR, nitrate reductase-like; NOS-like, NO synthase; PAO, polyamine oxidase; MSR, methionine sulfoxide reductase A and B.

ABA-NO interaction alleviates environmental stresses via changing the levels of gene expression. For example, at the transcript level, ABA and NO up-regulate the relative expression of MSR-related genes *OsMSRA4*, *OsMSRA5*, *OsMSRB1.1*, *OsMSRB3*, and *OsMSRB5* to prevent ROS from harming proteins and improve tolerance of NaCl in rice (Hsu and Lee, 2018; **Figure 2**). Furthermore, ABA inhibitor Flu and NO scavenger cPTIO can reverse the effects (Hsu and Lee, 2018). ABA relies on NO to notably up-regulate the expression of the antioxidant enzyme-related genes *TaSOD*, *TaCAT*, and *TaAPX* in wheat seedlings under heavy metal stress (**Table 2**; **Figure 2**). When ABA inhibitors are applied, their expression is down-regulated (Wu et al., 2018). Under low-light-induced oxidative stress, ABA also can improve levels of the antioxidant enzyme-related genes *POD*, *CnZn-SOD*, *Mn-SOD*, *CAT-A*, *CAT-B*, *APX2*, *APX4*, ABA receptor genes *FaPYR1* and *FaPYL1*, but *CAT-C* is down-regulation (Zhang et al., 2018b; **Table 2**; **Figure 2**). The authors further found that the expression of a NOS-associated gene *FaNOA1* is up-regulated by ABA and down-regulated by ABA inhibitor Flu, suggesting that ABA-mediated NO improves low-light tolerance by changing the expression of genes related to antioxidant enzymes (**Table 2**). Guo et al. (2014) report that the expression associated with PAs and ethylene biosynthesis gene *MfSAMS1* is significantly up-regulated by ABA, SNP, and H_2_O_2_ treatments in *Medicago* under low-temperature stress. The transcript levels of PAs biosynthesis-related genes including *PAO*, *CuAO1*, *CuAO2*, *SAMDC1*, *SAMDC2*, *SPDS1*, *SPDS2*, *SPMS*, antioxidant enzyme-related genes *Cu-SOD*, *Zn-SOD*, *CAT1*, *cAPX*, c*pAPX* are up-regulated in overexpression of *MfSAMS1* tobacco, indicating that *MfSAMS1* gene is improved low-temperature tolerance through up-regulating antioxidant enzymes and polyamine oxidation (Guo et al., 2014; **Table 2**; **Figure 2**). The exogenous Spd or Spm can up-regulate the expression of the NO-related gene *LeNR* and downregulation of *LeNOS* along with NO generation in tomato seedlings under low-temperature stress (Diao et al., 2017; **Table 2**). Furthermore, Put treatment can up-regulate the expression of the ABA biosynthesis gene *LeNCED1* as well as ABA production. When SNP is applied, *LeADC*, *LeADC1*, *LeODC*, and *LeSPDS* associated with polyamine biosynthesis are up-regulated (Diao et al., 2017; **Table 2**). The above results reveal that that PAs depend on ABA and NO to improve low-temperature tolerance by upregulating ABA and NO-related genes. When *CPK27* (calcium-dependent protein kinases) is silenced in tomato under low-temperature stress, the activities of NADPH oxidase, NR, and MPK1/2 are suppressed, and the NO, ABA, and H_2_O_2_ contents are decreased (Lv et al., 2018; **Table 2**; **Figure 2**). But when exogenous ABA is used, the effects are overturned, showing that *CPK27*-dependent ABA responds to low-temperature stress via elevating the activity of NADPH oxidase, NR, and generating NO, H_2_O_2_. Peng et al. (2022) indicate that the gene expression associated with flavonol biosynthesis pathways such as *C4H*, *CHS*, *CHI*, *F3H*, *F3’5’H*, *F3’H*, and *FLS* are significantly up-regulated in the leaves of creeping lilyturf under freezing stress. Likewise, ABA biosynthesis pathway gene expression levels, for example, *LUT5*, *ZEP*, *NCED*, *ABA2*, and *AAO3* are up-regulated as well as *VED* downregulation. In addition, NO content is increased, with *NOS* up-regulation and *pheophorbide a oxygenase* (*PAO)* down-regulation (**Table 2**; **Figure 2**). Exogenous ABA and NO enhance the levels of expression of genes related to NO biosynthesis *NtNOA1*, *NtNR2*, ABA biosynthesis *NtNCED1/3/4/5*, *NtAAO*, *NtSDR*, ABA signaling pathway *NtPYL2/6*, *NtPP2C*, *NtABF1/3*, and *NtSnRK2.2/2.3* to increase endogenous ABA and NO contents in *Nitraria tangutorum* seedlings under alkali conditions (Zhang et al., 2023). Moreover, the expression of genes involved in plasma membrane-localized Na^+^/H^+^ antiporter *NtSOS1*, vacuolar Na^+^/H^+^ antiporter *NtNHX1/2/3*, K^+^/H^+^ reverse transporter *NtKEA 3/5*, other ion transporter *NtKUP4*, *NtKCO*, and *NtHAK6/12* in roots are up-regulated by exogenous ABA and NO, following the down-regulation of the expression of *NtHKT1*, *NtHAK6/12*, *NtKCO*, and *NtKUP4* in leaves. The results show that exogenous ABA and NO can promote the increase of endogenous ABA and NO contents, which further regulate ion transporters to alleviate alkali tolerance. Even though these specific genes are involved in the interaction between ABA and NO, the regulatory network is not understood.

## 5 Conclusions and perspectives

Abiotic stresses present a great threat to the growth and development of plants. Growing attention is devoted to the role of interaction between ABA and NO in environmental stresses. The crosstalk between ABA and NO to alleviate multiple environmental stresses is discussed in this review, including drought, extreme temperature, salt, heavy metal, extreme light, UV-B, and alkali. ABA-NO crosstalk is potentially a strategy to enhance abiotic tolerance by modulating ROS, antioxidant enzymes, Pro, flavonoids, polyamines, ascorbate-glutathione cycle, water maintenance, photosynthesis, stomatal movement, and PTMs. Importantly, the review summarizes in detail that ABA-NO interaction can regulate some related genes to improve stress tolerance. These genes included those associated with MSR, antioxidant enzyme, ABA receptor, ABA biosynthesis, NO biosynthesis, PAs biosynthesis, calcium-dependent protein kinases, and flavonoid biosynthesis.

Although it is now known that crosstalk between ABA and NO plays a crucial role in conferring abiotic resistance in plants, many parts of this modulation are still missing. In the first place, the mechanisms of environmental perception by interaction between ABA and NO remain a topic of great interest. In addition, the regulatory mechanisms in multiple and simultaneous stresses need to be further explored. Thirdly, further studies are needed to investigate the signaling cascade mechanisms involved in abiotic and biotic stresses in plants, for example, Ca^2+^, K^+^, other signaling molecules, and plant hormones. Finally, the topic of NO-involved PTMs of specific proteins and specific sites is also a crucial subject. For the above, we will still face great challenges in solving these existential problems.

## Author contributions

JX: Writing – original draft, Writing – review & editing. XL: Visualization, Writing – original draft. YL: Visualization, Writing – original draft. WL: Visualization, Writing – original draft. ZW: Visualization, Writing – original draft. WY: Funding acquisition, Project administration, Supervision, Writing – review & editing. CL: Project administration, Supervision, Writing – review & editing. 
